# Laryngeal Aerodynamics in Children with Hearing Impairment versus Age and Height Matched Normal Hearing Peers

**DOI:** 10.1155/2013/394604

**Published:** 2013-07-18

**Authors:** Barshapriya Das, Indranil Chatterjee, Suman Kumar

**Affiliations:** ^1^AYJNIHH, ERC, Kolkata 700090, India; ^2^Department of Speech Language Pathology, AYJNIHH, ERC, BT Road, Bon Hooghly, Kolkata 700090, India

## Abstract

Lack of proper auditory feedback in hearing-impaired subjects results in functional voice disorder. It is directly related to discoordination of intrinsic and extrinsic laryngeal muscles and disturbed contraction and relaxation of antagonistic muscles. A total of twenty children in the age range of 5–10 years were considered for the study. They were divided into two groups: normal hearing children and hearing aid user children. Results showed a significant difference in the vital capacity, maximum sustained phonation, and fast adduction abduction rate having equal variance for normal and hearing aid user children, respectively, but no significant difference was found in the peak flow value with being statistically significant. A reduced vital capacity in hearing aid user children suggests a limited use of the lung volume for speech production. It may be inferred from the study that the hearing aid user children have poor vocal proficiency which is reflected in their voice. The use of voicing component in hearing impaired subjects is seen due to improper auditory feedback. It was found that there was a significant difference in the vital capacity, maximum sustained phonation (MSP), and fast adduction abduction rate and no significant difference in the peak flow.

## 1. Introduction

Lack of proper auditory feedback in hearing-impaired subjects results in functional voice disorder. It is directly related to discoordination of intrinsic and extrinsic laryngeal muscles and disturbed contraction and relaxation of antagonistic muscles. Generally, the voice organ of a hearing-impaired child shows no anatomical abnormalities in the first years of life. Data in the literature suggest that the impairment of the voice organ is a secondary effect and results not only from abnormal auditory feedback but also from inappropriate hearing, voice, and speech rehabilitation in early childhood, or even its cessation [1, 2]. 

Research on the physiopathology of voice and speech in hearing-impaired children proves that a physiological deficiency of the larynx affects phonation and articulation. Specialists therefore emphasize that voice and speech rehabilitation should begin as soon as possible, helping to maintain correct functioning of the larynx [3, 4]. Changes in the larynx of a prelingually hearing-impaired child and changes in his/her voice develop in the first years of life. The nature of these disorders depends on numerous conditions, including degree and type of hearing loss, duration of the hearing impairment, the moment of its occurrence, the treatment applied, and benefits obtained, as well as the effectiveness of wide-range rehabilitation and other environmental factors [5]. Limited functioning of the patient's ear impairs their voice control and may have a negative effect on their communication. Differences between the vocalizations of normal-hearing and hearing-impaired infants do emerge at an early age, but the differences are seen in phonemic production rather than quantity of vocal output as suggested by Mavilya [6].

## 2. Need of Study

Early intervention of hearing impairment has brought a revolution in our society in helping the hearing-impaired children to acquire speech and language utilizing their critical age. Hence, the development of speech and language in hearing children depends on the age of intervention. There is literature which supports the differences in voice quality in normal and hearing-impaired persons both acoustically and aerodynamically. But still, there are poor attempts in studying the voice quality of hearing aid user children and normal children in Indian population. So, there is a need to compare the laryngeal aerodynamic parameters in normal and hearing-impaired children, which will help the speech language pathologist to monitor the changes in the voice quality in pre- and posttherapeutic process. This study will also show light to the correlation between speech and language development with the voice quality of the hearing-impaired children.

## 3. Aim and Objective

To compare the laryngeal aerodynamic parameters in age matched hearing aid user children with normal children.

## 4. Method

### 4.1. Participants

A total of 20 children in the age range of 5–10 years, with a mean age of 8 years (SD = 1.5 years) and with an average height of 118.4 cm (SD = 2.32 cm), were considered for the study. They were divided into two groups: normal and hearing aid user children, with 10 children in each group.

### 4.2. Inclusion Criteria

The hearing aid user children had bilateral profound sensorineural hearing loss, used multichannel binaural digital hearing aids, comprehend and express simple sentence, and followed bidirectional verbal instruction.

### 4.3. Exclusion Criteria

Children having chronic medical problems, history of voice problems or laryngeal pathology, or any respiratory diseases for the last 3 months and syndromic children were excluded from the study.

### 4.4. Equipment Used

- Aerophone II (voice function analyzer) (manufacturer F-J Electronics, Vedbaek, Denmark) was used for assessing the laryngeal aerodynamic properties. It takes the advantage of a sophisticated combination of a hardware transducer system with transducers recording airflow, air pressure, and acoustic signal and a computerized data processing. All electronics including the microprocessor and the transducers are miniaturized and built into a small box mounted in the holder for handle and mask.

#### 4.4.1. Calibration of Equipment. 

Calibration of the equipment was done as per Aerophone II manual (2005).

- Calibration of air pressure: a calibration factor for the air pressure is not necessary as it is fairly stable and not influenced by patients' responses. The air pressure transducer is adjusted from the factory and does not need any further adjustment.
- For calibrated SPL recordings from aerophone, the microphone is factory tested (by means of Bruel & Kjaer, Integrating Precision Sound Level Meter, type 2230) and the appurtenant calibration factor is 0.72. If no calibration factor is typed when the programme asks for a calibration factor to ensure the SPL values are exact (within ±0.2 dBSPL), a default calibration factor is used which is an average of several microphone calibration factors as measured by F-J Electronics. To ensure that the microphone measures correctly, the microphone was pushed so far through the PVC rubber sleeve that all the side holes are free. Microphone positions for range
   30–80 dB in flow head,
   50–100 dB in flow head,
   70–120 dB in flow head.
- For airflow calibration, 1 liter calibration syringe (accuracy ±0.5%, calibrated at 20°C) was taken. While calibrating the approximately one second airflow for big flow head and approximately four seconds for the small flow head was taken. The piston of the syringe was pressed with a constant and a stable movement during the calibration. The calibration was started with a coarse calibration making 3–5 measurements with the 1 liter syringe. When the result was within 1-2% from the correct value, a new calibration with several successive recordings was done. The programme calculated the average calibration factor of the recordings made, which is stored in the setup file. The same procedure was repeated for both flow heads.
- Warm-up time: the airflow and pressure transducers need approximately 15 minutes in order to obtain a stable zero line after the system has been turned on. After that time the auto zeroing circuit is able to keep the zero line stable during normal recording sessions, which normally do not exceed 60 seconds. When not recording, the system is constantly auto zeroing.
- Room temperature: the room temperature was kept at 29°C which was measured by the digital thermometer provided in the instrument suitcase. It was recommended to keep the room temperature between 10°C and 40°C.
- Viscosity of air: when the expired air passes the gauze in the flow head, it has a temperature of 35°C as the air is cooled 2°C below body temperature when passing the speech organs and the flow head tubes. The software takes the changed viscosity of the expired air into account. Table 1 relates the viscosity of air to the temperature.

### 4.5. Procedure

Four aerodynamic parameters were taken for assessment: peak flow, vital capacity, maximum sustained phonation, and fast adduction abduction rate. For the measurement of the parameters, separate instructions for separate parameters were demonstrated and repeated to the subjects till they get thorough about it. Throughout the data registration, the height and posture of the client were considered and care was taken to fix the mask and cardboard tube of aero phone properly, so that no air leakages happen.

Following procedures were used to measure the following parameters.

*(1) Vital Capacity*

- The following setting was made in the programme as per the instruction given in the manual which were kept constant for all subjects. Flow head F1000LS was used for the registration with pressure setting of flow range 0–10 l/sec.
- The subject was asked to inhale as deep as possible and then to exhale all the airs through the mouth tube until the lungs are completely empty. The registration was made in standing position. It was told to take care that the lips close airtight round the disposable mouth tube. The nose clamp was used during the registration. The instructions were repeated whenever needed and demonstrations were made.
- The subject exhaled into the mouth tube and the data was stored in the computer. Each subject was asked to give three trials and the highest was considered as the vital capacity for that subject. Thus the vital capacity and its duration were calculated.

*(2) Peak Flow*

- The following setting was made in the programme as per the instruction given in the manual which were kept constant for all subjects.
- Flow head F1000LS was used for the registration with pressure setting of flow range 0–10 l/sec.
- The subject was asked to inhale as deep as possible and then to exhale all the air through the mouth tube as fast and as strong as possible. The registration was made in standing position. It was told to take care that the lips close airtight round the disposable mouth tube. The nose clamp was used during the registration. The instructions were repeated whenever needed and demonstrations were made.
- The subject exhaled into the mouth tube and the data was stored in the computer. Each subject was asked to give three trials and the highest value was considered for that subject. Thus the peak flow, forced volume 1 second and Duration was calculated from the result.

*(3) Maximum Sustained Phonation*

- The following setting was made in the programme as per the instruction given in the manual which were kept constant for all subjects.
- Flow head F 300LS was used with pressure setting of 5.0 l/s and 50–100 dB was selected from the SPL menu.
- As the subjects taken, consisted of males, hence the pitch level was set at 131 Hz, with an intensity range of 75 dB.
- The subject was asked to fix the mask covering mouth and nose over the face and was asked to take care that there was no leakage through the mask during the measurement.
- The subjects were instructed to take a deep breath and try to produce a matching tone produced by the computer, maintaining its loudness. They could use the indicator (computer monitor) to maintain the loudness. The subjects were asked to say /a/ as long as and as comfortable as possible.
- The instructions were repeated whenever needed and demonstrations were made.
- After the phonation, the data were stored in the computer.

*(4) Fast Abduction-Adduction Rate*

- The following setting was made in the programme as per the instruction given in the manual which were kept constant for all subjects.
- Flow head F300LS was used with pressure setting of 1.00 l/s and intensity range was 30–80 dB close.
- The subject was asked to fix the mask covering mouth and nose over the face and was asked to take care that there was no leakage through the mask during the measurement.
- The subjects were instructed to say “*ah ah ah ah*” as fast as possible after taking in deep breath. The voiced production was recorded.
- The instructions were repeated whenever needed and demonstrations were made.
- After the recording, the data of volume, mean airflow rate, and adduction abduction rate were stored in the computer.

## 5. Results and Discussion

### 5.1. Results

#### 5.1.1. Peak Flow

The aerodynamic scores analysis for peak flow for normal children showed a mean value of 1.4682 litre/sec, (SD = 0.386 litre/sec) as compared to hearing aid user children, who showed a mean value of 1.202 litre/sec (SD = 0.348 litre/sec) (Table 2).

Independent *t*-test results showed no significant difference in the peak flow value with (*t* (18) = 1.618, *P* = 0.123) being statistically significant at 95% level of confidence (Table 3).

#### 5.1.2. Vital Capacity

The aerodynamic scores analysis for vital capacity for normal children showed a mean value of 0.7103 litre/sec (SD = 0.091 litre/sec) as compared to hearing aid user children, who showed a mean value of 0.454 litre/sec (SD = 0.058 litre/sec) (Table 4).

Independent *t*-test results showed a significant difference in the vital capacity (*t* (17) = 7.153, *P* = 0.00), being statistically significant at 95% level of confidence (Table 5).

#### 5.1.3. Maximum Sustained Phonation

The aerodynamic scores analysis for maximum sustained phonation for normal children showed a mean value of 9.716 seconds, (SD = 0.419 seconds) as compared to hearing aids user children, who showed a mean value of 4.327 seconds (SD = 0.736 seconds) (Table 6).

Independent *t*-test results showed a significant difference in the maximum sustained phonation (*t* (18) = 20.096, *P* = 0.00) being statistically significant at 95% level of confidence (Table 7).

#### 5.1.4. Fast Adduction Abduction Rate

The aerodynamic scores analysis for fast adduction abduction rate for normal children showed a mean value of 7.763 cycles/second (SD = 0.181 cycles/second), respectively, as compared to hearing aid user children, who showed a mean value of 5.790 cycles/second (SD = 3.122 cycles/second) (Table 8).

Independent *t*-test results showed a significant difference in the fast adduction abduction rate for normal and hearing aid user children with (*t* (18) = 7.050, *P* = 0.00) being statistically significant at 95% level of confidence (Table 9).

### 5.2. Discussion

The present finding indicates that the aerodynamic parameters of voice of hearing aid user children differ significantly in terms of vital capacity, maximum sustained phonation (MSP), and fast adduction abduction rate (Figure 5). Although no significant difference was found for the Peak flow, a reduced value for this parameter was seen for the hearing aid user children.

**Figure 1:**
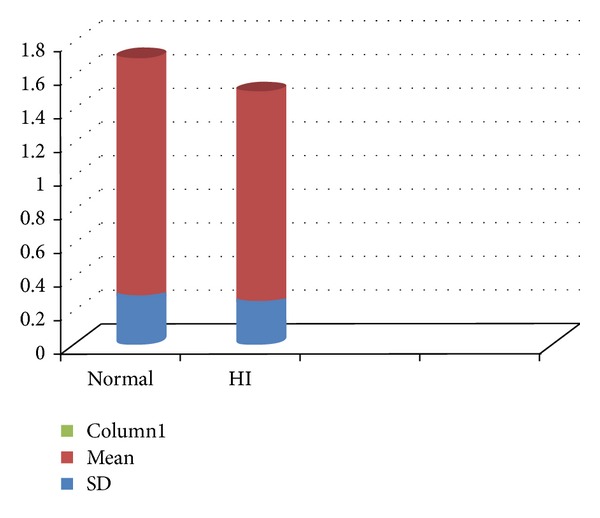
Comparison of mean and standard deviation for peak flow (in litre/sec) between normal children and hearing aid user children.

 Peak flow otherwise known as peak expiratory flow (PEF) is the maximum flow achieved during an expiration delivered with maximal force starting from the level of maximal lung inflation. Measurements of PEF are of value in identifying airflow limitation [7]. An absence of significant difference in peak flow for both groups suggests the presence of physiologically healthy and functional lungs for the airflow supply that will be required for speech production (depicted in Figure 1). This finding is supported by Forner and Hixon [8] which showed that the mechanical adjustments of the respiratory mechanism in preparing to speak (i.e., the relative posture of the rib cage versus the abdomen) in profoundly hearing-impaired speakers were often correct.

**Figure 2:**
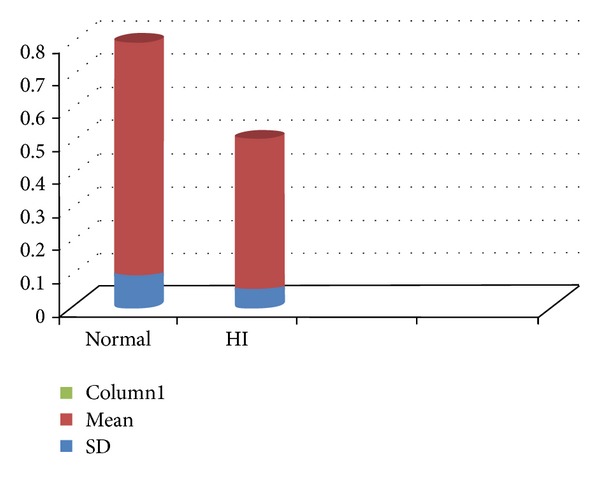
Comparison of mean and standard deviation for vital Capacity (in litre/sec) between normal children and hearing aid user children.

Vital capacity is the total amount of air that can be exhaled after a maximum inspiration [9]. A reduced vital capacity in hearing aid user children suggests a limited use of the lung volume for speech production. Studies on the respiratory patterns of profoundly hearing-impaired speakers have shown that they evidence at least two kinds of problems. The first is that they initiate phonation at too low a level of vital capacity and also that they produce a reduced number of syllables per breath [8, 10, 11], the second problem is that they mismanage the volume of air by inappropriate valving at the laryngeal level. These findings correlate our findings of poor vital capacity in hearing-impaired children (depicted in Figure 2).

**Figure 3:**
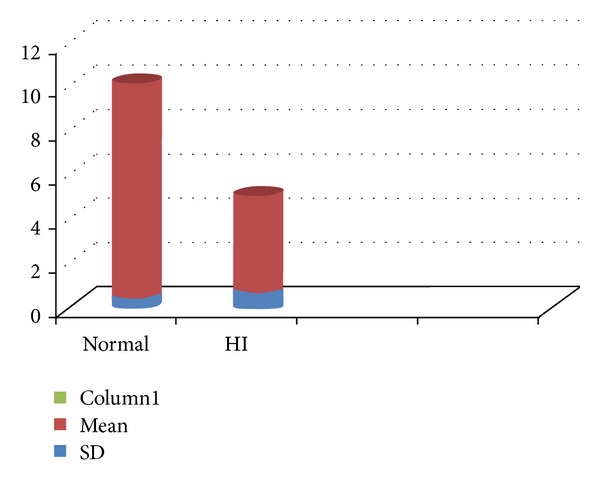
Comparison of mean and standard deviation for maximum sustained phonation (in seconds) between normal children and hearing aid user children.

Maximum sustained phonation is considered a maximum performance task which measures a person's ability to efficiently manage an adequate air supply during phonation [12]. MSP is a helpful clinical tool to estimate vocal proficiency [13, 14]. In this study, it can be derived that the hearing aid user children have poor vocal proficiency which is reflected in their voice (depicted in Figure 3).

**Figure 4:**
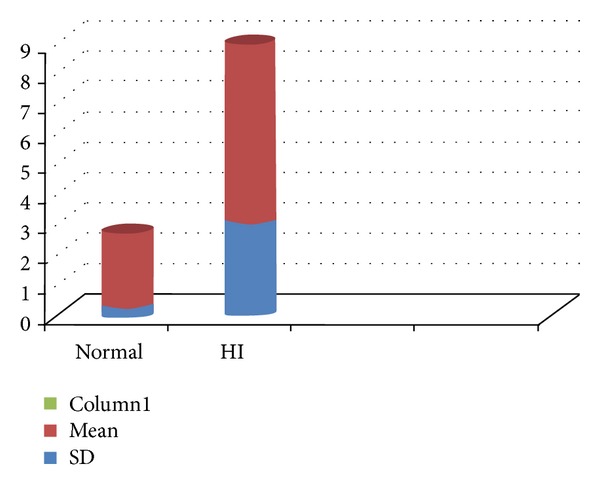
Comparison of mean and standard deviation for fast adduction abduction rate (in litre/sec) between normal children and hearing aid user children.

**Figure 5:**
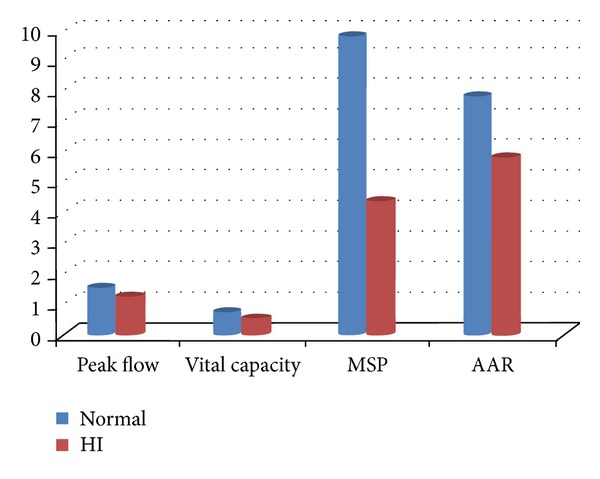
Comparison of mean value of scores obtained for peak flow (in litre/sec), vital capacity (in litre/sec), maximum sustained phonation (in seconds), and Adduction Abduction Rate (in litre/sec) in normal and hearing aid user children.

The parameter fast adduction abduction rate indicates the rate of closing and opening movements of vocal fold in movements per second. The pronunciation may be either voiced or voiceless, but the result will differ from each other in each case. The use of voicing component in hearing-impaired subjects is seen due to improper auditory feedback. Hence, a difference in the adduction abduction rate is observed in the hearing aid users. Forner and Hixon [8] reported that hearing-impaired speakers paused at inappropriate linguistic boundaries either to inspire or alternatively to waste air, and thus they produced fewer syllables per breath unit. Hearing-impaired speakers were also found to initiate phonation at inappropriate lung volumes and to speak within a fairly restricted lung volume range. These explanations correlate with our findings of poor fast adduction abduction rate in hearing-impaired children (depicted in Figure 4). Monsen et al. [15] reported that an individual glottal pulse for a hearing-impaired speaker was not abnormal but that differences between hearing-impaired and hearing subjects were seen for successive changes of the glottal waveform from one period to another. Glottal waveforms of hearing-impaired speakers also showed evidence of diplophonia and creaky voice. Thus, the authors hypothesized that hearing-impaired speakers have difficulty controlling overall tension of the vocal folds and also subglottal pressure. Secondly, high-speed laryngeal films have also provided evidence of abnormal laryngeal function in hearing-impaired speakers [16]. Several profoundly hearing-impaired speakers show evidence of inappropriate positioning of the vocal folds prior to the onset of phonation and subsequent patterns of abnormal vocal fold vibration.

Various findings have been documented in the literature regarding the laryngeal aerodynamic characteristics of hearing-impaired children. Although the basis of breathing anomalies and abnormal pausing in these cases are not understood, it could be related to aberrant functioning at all levels of the speech mechanism including the respiratory, phonatory, articulatory, and resonatory speech subsystems. Compared to speakers with normal hearing, speakers with bilateral congenital profound hearing impairment tend to initiate speech at a level of lung volume that is either too high or too low and to speak into functional reserve capacity; moreover, the mean airflow per syllable is twice that of speakers with normal hearing which is reflected in our findings of reduced vital capacity and adduction abduction rate. Profoundly hearing-impaired speakers also demonstrated more aberrant rates of oral airflow than did normal-hearing (NH) and severe hearing-impairment (SHI) speakers. Consequently, these speakers produced fewer syllables per breath group and often paused for inspiration at grammatically inappropriate locations. The authors concluded that the disorders in the speech respiratory behaviors resulted from the combined influences of poor respiratory control, incoordination between larynx and upper airway, and/or failure to follow grammatical phrasing.

Laryngeal dysfunction has been documented in speakers with hearing impairments. Using transillumination to monitor glottal activity and an electrical transconductance technique to record temporal patterns of oral articulation, McGarr and Löfqvist [17] studied 3 adults with SHI and observed inappropriate abduction gestures between words for all their talkers. Metz et al. [18] also observed abnormal abductory behaviors in 4 adults with profound hearing impairment, which appeared to result in air wastage during speech production. Based on a comprehensive speech/voice physiological assessment of 14 adults with hearing impairment, Higgins et al. [19] observed both hypo- and hyperadduction of the vocal folds, although hyperadduction was more common. Both patterns have the potential to significantly affect speech breathing behavior.

Aberrant pausing in SHI may also be due to aberrant articulatory and velopharyngeal behavior. Specifically, SHI speakers tend to speak slower and exhibit more nasality than NH speakers. Slowed speech is probably associated with increased pauses as speakers need to replenish their air supply more frequently. Similarly, air escape from the velopharyngeal port during speech could diminish the air supply for speech, resulting in more frequent inspirations. The purpose of this investigation is to identify the potential components of the abnormal pausing patterns. This information will serve as a basis for more in-depth investigations into speech subsystem changes that might account for the observed behavior.

Speech breathing anomalies in speakers with SHI have commonly been characterized using kinematic or aerodynamic technology. It has been shown that speakers with SHI exhibit more inappropriate inspiratory loci; in addition, they tend to inspire at nongrammatical locations when reading passages, apparently because of improper respiratory control. Aberrant pausing patterns could affect its temporal breath group structure, speaking rate within breath groups, and speech intelligibility. 

## 6. Conclusion

Correlations between different heights of the children and aerophone parameters should also be established for the hearing-impaired children with respect to their normal hearing counterparts. This information will serve as a basis for more in-depth investigations into speech subsystem changes that might account for the observed behavior.

## Figures and Tables

**Table 1:** Relation between viscosities of air to the temperature (Aerophone II manual, 2005).

Temperature in °C	0	10	20	30	40
Viscosity in Cp	170.9	175.9	180.8	185.6	190.4

**Table 2:** Mean and standard deviation values of peak flow (in litre/sec), for normal and hearing impaired children.

Subjects	Mean (in litre/sec)	Std. deviation
Normal children	1.4682	0.38596
Hearing impaired children	1.2022	0.34819

**Table 3:** Independent *t*-test for peak flow, for normal children and hearing aid user children.

Parameter	Mean difference (in litre/sec)	Std. error difference	*t*	Df	Sig. (2-tailed)
Peak flow	0.26600	0.16438	1.618	18	0.123

**Table 4:** Mean and standard deviation values of vital capacity (in litre/sec), for normal and hearing impaired children.

Subjects	Mean (in litre/sec)	Std. deviation
Normal	0.7103	0.09151
HI	0.4544	0.05879

**Table 5:** *t*-Test for vital capacity, for normal children and hearing aid user children.

Parameter	Mean difference (in litre/sec)	Std. error difference	*t*	Df	Sig. (2-tailed)
Vital capacity	0.25586	0.03577	7.153	17	0.000

**Table 6:** Mean and standard deviation values of maximum sustained phonation (in seconds), for normal and hearing impaired children.

Subjects	Mean (in seconds)	Std. deviation
Normal	9.7165	0.41983
HI	4.3274	0.73682

**Table 7:** *t*-Test for maximum sustained phonation, for normal children and hearing aid user children.

Parameter	Mean difference (in seconds)	Std. error difference	*t*	Df	Sig. (2-tailed)
Maximum sustained phonation	5.38910	0.26817	20.096	18	0.000

**Table 8:** Mean and standard deviation values of peak flow (in litre/sec), for normal and hearing impaired children.

Subjects	Mean (in litre/sec)	Std. deviation
Normal	7.7639	0.18121
HI	5.7904	3.12260

**Table 9:** Independent *t*-test for fast adduction abduction rate (in litre/sec), for normal children and hearing aid user children.

Parameter	Mean difference (in litre/sec)	Std. error difference	*t*	Df	Sig. (2-tailed)
Fast adduction abduction rate	6.97350	0.98911	7.050	18	0.000
